# Sinonasal Delivery of Resveratrol via Mucoadhesive Nanostructured Microparticles in a Nasal Polyp Mouse Model

**DOI:** 10.1038/srep40249

**Published:** 2017-01-10

**Authors:** Mingyu Lee, Chun Gwon Park, Beom Kang Huh, Se-Na Kim, Seung Ho Lee, Roza Khalmuratova, Jong-Wan Park, Hyun-Woo Shin, Young Bin Choy

**Affiliations:** 1 Obstructive Upper airway Research (OUaR) Laboratory, Department of Pharmacology, Seoul National University College of Medicine, Seoul, 03080, Republic of Korea; 2 Department of Biomedical Science, Seoul National University Graduate School, Seoul, 03080, Republic of Korea; 3 Cancer Research Institute, Seoul National University College of Medicine, Seoul, 03080, Republic of Korea; 4 Institute of Medical & Biological Engineering, Medical Research Center, Seoul National University, Seoul, 03080, Republic of Korea; 5 Interdisciplinary Program in Bioengineering, College of Engineering, Seoul National University, Seoul, 08826, Republic of Korea; 6 Ischemic/Hypoxic Disease Institute, Seoul National University College of Medicine, Seoul, 03080, Republic of Korea; 7 Department of Otorhinolaryngology-Head and Neck Surgery, Seoul National University Hospital, Seoul, 03080, Republic of Korea; 8 Department of Biomedical Engineering, Seoul National University College of Medicine, Seoul, 03080, Republic of Korea

## Abstract

Resveratrol (RSV) has been shown to effectively suppress chronic rhinosinusitis with nasal polyps in a mouse model; however, when locally administered to the sinonasal cavity, bolus RSV is limited by low drug bioavailability owing to its low aqueous solubility and relatively rapid clearance from the administration site. To address this limitation, we propose mucoadhesive nanostructured microparticles (PLGA/PEG NM) as a potential carrier for the sinonasal delivery of RSV. In this study, PLGA/PEG NM released RSV in a sustained manner. Owing to the enlarged specific surface area of the nanostructures, PLGA/PEG NM had synergistically enhanced mucoadhesiveness and thus showed improved *in vivo* retention properties in the sinonasal cavity. Therefore, when tested in a mouse nasal polyp model, PLGA/PEG NM mitigated polyp formation and restored epithelial integrity better than the control treatments. The therapeutic effect was similar at half the dose of PLGA/PEG NM, suggesting improved local bioavailability of RSV in the sinonasal cavity.

Chronic rhinosinusitis with nasal polyps is a Th2-skewed heterogeneous inflammatory disease that is often accompanied by prominent eosinophilic infiltration, an increase in subepithelial thickness and the formation of microcavities in the mucosal epithelium^1^,^2^. Even after treatment with the current optimal regimen of steroids or with functional endoscopic sinus surgery (FESS), the overall recurrence rates often exceed 25%, varying with history of allergy or asthma and age^3^,^4^. Recently, we showed that hypoxia-inducible factor-1 (HIF-1) can induce the epithelial-to-mesenchymal transition (EMT) of the nasal polyp epithelium, leading to nasal polypogenesis^5^. Thereafter, we reported that Sirtuin 1 (SIRT1) can be considered a novel therapeutic target because of its suppression of HIF-1α-induced mucosal remodeling; thus, we proposed a SIRT1 activator, resveratrol, as a novel therapeutic agent^6^. Resveratrol (RSV), a naturally occurring polyphenolic compound, has received great attention for its anti-carcinogenic, anti-inflammatory and anti-oxidant effects^7^,^8^,^9^. Despite its outstanding anti-polyp effects in mouse models, the bioavailability of RSV is low when it is locally delivered to the nasal cavity in solutions or suspensions^10^,^11^. The utility of RSV is limited by its low aqueous solubility and its susceptibility to rapid clearance from the nasal cavity. Therefore, repeated intranasal administrations are often required to achieve a therapeutic effect for a prolonged period^12^,^13^.

To overcome this limitation, we propose mucoadhesive microparticles with a nanostructured surface as carriers for the nasal delivery of RSV. The microparticles are mainly composed of poly (lactic-co-glycolic acid) (PLGA) to allow the sustained delivery of RSV^14^,^15^. A mucoadhesive polymer, polyethylene glycol (PEG)^16^,^17^, was used as an additional constituent to increase microparticle residence time in the mucosal layer of the nasal cavity^18^. Together with PEG, the nanostructuring of the surface of microparticles, which provided an enlarged specific surface area, led to a synergistic increase in residence time. Therefore, we hypothesized that RSV-loaded microparticles might reside in the nasal cavity for a prolonged time and release RSV in a sustained manner, thereby enhancing its bioavailability and yielding a high potential for resolving the refractory clinical problems described above. To our knowledge, this is the first study to report mucoadhesive, nanostructured microparticles as sinonasal delivery carriers for RSV and to evaluate their *in vivo* efficacy for the treatment of chronic rhinosinusitis in animal nasal polyp models.

In the present study, mucoadhesive nanostructured microparticles (PLGA/PEG NM) were fabricated based on the method reported in our previous study^19^, wherein a solution containing both PLGA and PEG was electrospun to produce a nanofibrous sheet that was then freeze-milled to yield microparticles with a nanostructured morphology. As controls, we also prepared three other distinct types of microparticles: spherical microparticles without mucoadhesion (i.e., spherical microparticles with PLGA only; PLGA MS), spherical microparticles with mucoadhesion (i.e., spherical microparticles with a blend of PLGA and PEG; PLGA/PEG MS) and nanostructured microparticles without mucoadhesion (i.e., nanostructured microparticles with PLGA only; PLGA NM).

The microparticles were loaded with a fluorescent marker, diethylthiatricarbocyanine iodide (DTTCI) to examine their retention properties in the sinonasal cavity; after nasal delivery, the quantity of remaining microparticles was evaluated via *in vivo* fluorescence imaging. The RSV-loaded microparticles were assessed via *in vitro* drug-release experiments to examine their sustained drug-release properties. We used a mouse model of eosinophilic rhinosinusitis with nasal polyps to investigate the therapeutic effect *in vivo*^10^. The PLGA/PEG NM and RSV solutions were each delivered sinonasally, and the degree of disease suppression was verified using Sirius red staining, Giemsa staining, Masson’s Trichrome staining and Periodic Acid–Schiff (PAS) staining to evaluate eosinophils, goblet cells, collagen deposition and mast cells, respectively.

## Results

### Microparticle Characterization

The microparticles were first imaged with a scanning electron microscope, as shown in Figure S1 in the Supplementary Information. The MS prepared by the conventional emulsion method exhibited a spherical shape and a smooth surface, whereas the NM showed a rough nanofibrous surface^19^,^20^,^21^. The overall morphologies of the MS and NM were not influenced by the presence of PEG, RSV or DTTCI, in accordance with our previous study^19^,^20^,^21^. Here, the MS and NM were prepared so that they possessed a similar size distribution, which was measured to be 6.72–7.64 μm (Figure S2 and Table S1 in the Supplementary Information). The actual amount of PEG in the microparticles was determined with a proton ^1^H - nuclear magnetic resonance (^1^H NMR) spectrometer; the PEG content was measured to be 9.95 and 10.17% for PLGA/PEG MS and PLGA/PEG NM, respectively (Table S1). According to CO_2_ adsorption/desorption analysis, the specific surface area of the NM was more than 10 times larger than that of the MS because of the rough nanostructure on the surface of the NM. The specific surface areas were measured to be 291.23 m^2^/g and 28.88 m^2^/g for NM and MS, respectively. Regardless of microparticle type, similar amounts of DTTCI and RSV were loaded, showing ranges of 7.93–8.87 μg/mg and 1.06–1.78 μg/mg, respectively (Table S2). To assess the crystallinity of RSV in microparticles, we assessed the powder X-ray diffraction (PXRD) patterns of intact PLGA, intact PEG, intact RSV and RSV loaded PLGA/PEG NM, which suggested that RSV was distributed in the PLGA/PEG NM without forming a crystalline structure (Figure S3 in the Supplementary Information). To test the mucoadhesive property, we compared the binding capacity of mucin to the PLGA NM and PLGA/PEG NM under *in vitro* environment^22^. Our result revealed that more mucin was adsorbed to the PLGA/PEG NM (81.96 ± 1.86 μg per mg microparticles) than to the PLGA NM (70.13 ± 2.18 μg per mg microparticles). This could be ascribed to the presence of a mucoadhesive polymer, PEG, in the PLGA/PEG NM^17^.

**Figure 1:**
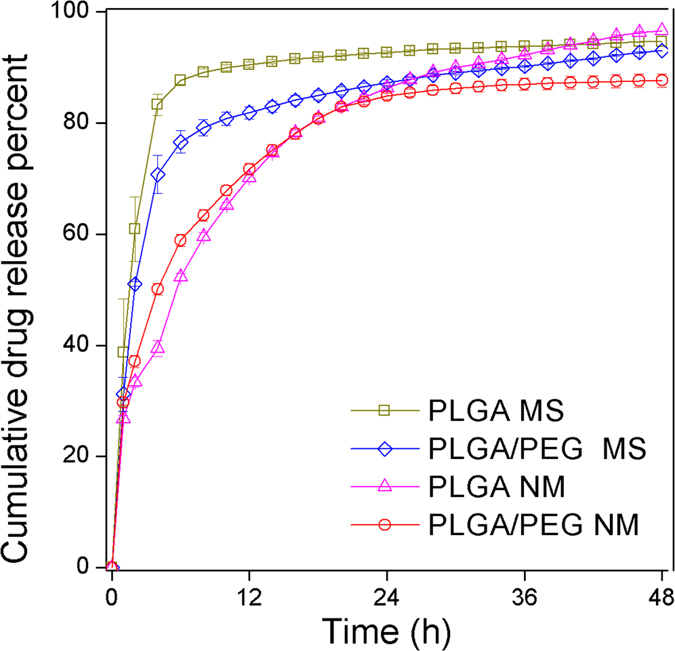
*In vitro* release profiles of RSV from the microparticles. The experiment was performed in PBS (pH 7.4) containing 1% w/v Tween 20. At defined time points, the release medium was collected and measured by HPLC.

For all microparticle types, we examined the *in vitro* release profile of RSV in phosphate buffered saline (PBS) at pH 7.4 and 37 °C, as shown in Fig. 1. RSV was released from all of the microparticles in a sustained manner. For PLGA MS and PLGA/PEG MS, 87.7 and 76.6% of the loaded RSV was released, respectively, during the first 6 h; subsequently, 7.0–16.4% of the RSV was slowly released during the next 42 h. For PLGA NM and PLGA/PEG NM, 52.3 and 58.9% of the loaded RSV was released, respectively, during the first 6 h, while 28.7–44.3% of the RSV was released over the next 42 h. Although not significantly different, however, a slightly higher release of RSV was expected in the fluid at pH 5.5 in nasal cavity (Figure S4 in the Supplementary Information). Under this condition, RSV is known to be more stable^11^ and thus, more effective RSV would be available. The difference between the MS and NM in the size of the initial burst release may be ascribed to their different preparation methods. For the MS, much of the initially loaded RSV can be lost during the emulsion process, which would leave many pores or conduits inside the microparticles that would enhance the release of the encapsulated RSV^23^,^24^. Meanwhile, the NM were prepared under a dry environment via electrospinning and milling, possibly yielding a more dense polymer matrix structure in the microparticles. This effect appeared to be more dominant than that of the specific surface area with respect to drug release.

### *In Vivo* Sinonasal Retention Properties

**Figure 2:**
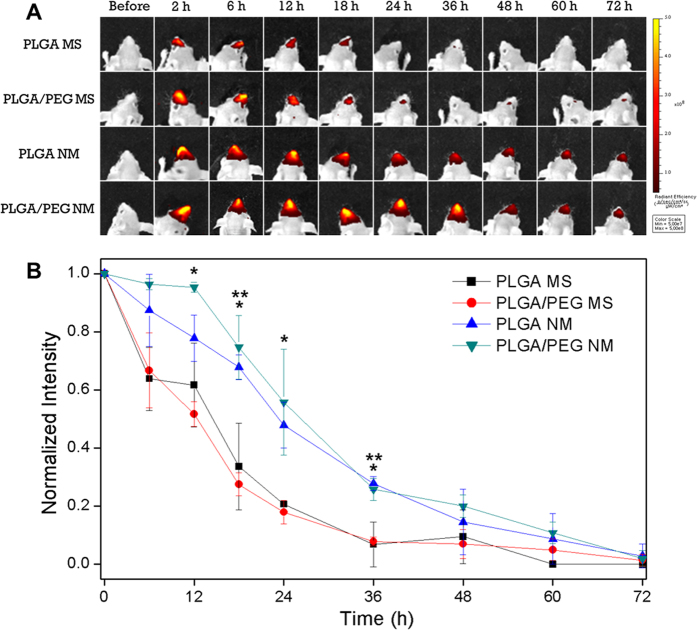
*In vivo* sinonasal retention profiles of microparticles. (**A**) Fluorescence images of the head were obtained using an *in vivo* imaging system at defined times after sinonasal administration of DTTCI-loaded microparticle suspensions to male BALB/c mice. (**B**) The fraction of microparticles remaining in the sinonasal cavity was determined by measuring the intensity of the fluorescence signal in the nasal cavity in comparison to the maximal intensity obtained 2 h after administration. ^*^At 12, 18, 24 and 36 h, PLGA/PEG NM were significantly different from PLGA MS and PLGA/PEG MS (*P* < 0.05). ^**^At 18 and 36 h, PLGA NM were significantly different from PLGA MS and PLGA/PEG MS (*P* < 0.05).

To examine the sinonasal retention properties of the microparticles, they were loaded with DTTCI, a near-infrared fluorescent dye, instead of RSV and were administered intranasally to living animals. We measured the amount of microparticles remaining in the nasal cavity over time using an *in vivo* imaging system (IVIS). DTTCI is known to be almost insoluble in water; thus, its release from the microparticles was expected to be minimal under our experimental conditions. As shown in Fig. 2, the MS were cleared relatively rapidly from the sinonasal cavity. Regardless of the presence of PEG, 64–67% of the MS remained 6 h after administration, while less than 20% of the MS were observed after 24 h. Thus, the half-lives of microparticle residence in the sinonasal cavity were calculated as 10.5 ± 2.8 and 9.1 ± 0.3 h for PLGA MS and PLGA/PEG MS (Table 1), respectively.

In contrast, the PLGA NM exhibited an apparent increase in sinonasal retention. Of the initially administered PLGA NM, 87% remained at 6 h, 78% remained at 12 h and 48% remained 24 h after administration. The half-life of PLGA NM was 21.5 ± 2.5 h (Table 1), representing a more than 2-fold increase when compared with that of PLGA MS and PLGA/PEG MS. This increase in sinonasal retention may be ascribed to van der Waals forces resulting from the enlarged specific area of the NM, which appeared to improve microparticle adhesion to the surface of the sinonasal space. Thus, the sinonasal retention of PLGA NM was significantly different from that of PLGA MS and PLGA/PEG NM at 18 h and 36 h (*P* < 0.05).

PLGA/PEG NM, i.e., the microparticles with both a nanostructured surface and mucoadhesiveness, exhibited the greatest sinonasal retention. More than 96% of these microparticles remained at 6 h, and their subsequent clearance occurred at a slow rate, as approximately 95, 75 and 56% of the microparticles remained in the sinonasal cavity at 12, 18 and 24 h, respectively. The half-life of PLGA/PEG NM was 23.5 ± 4.7 h (Table 1), which was the highest among the microparticle types tested in this study. Compared with PLGA MS and PLGA/PEG MS, the retention of PLGA NM showed significant differences at 12, 18, 24 and 36 h (*P* < 0.05). This considerable improvement in sinonasal retention may be attributed to synergistically improved mucoadhesiveness due to the enlarged specific surface area originating from the nanostructure^19^,^20^,^21^; thus, the PLGA/PEG NM used here better adhered to the mucosal layer in the sinonasal space.

### *In Vivo* Effects on Nasal Polyps and Epithelial Disruption

**Figure 3:**
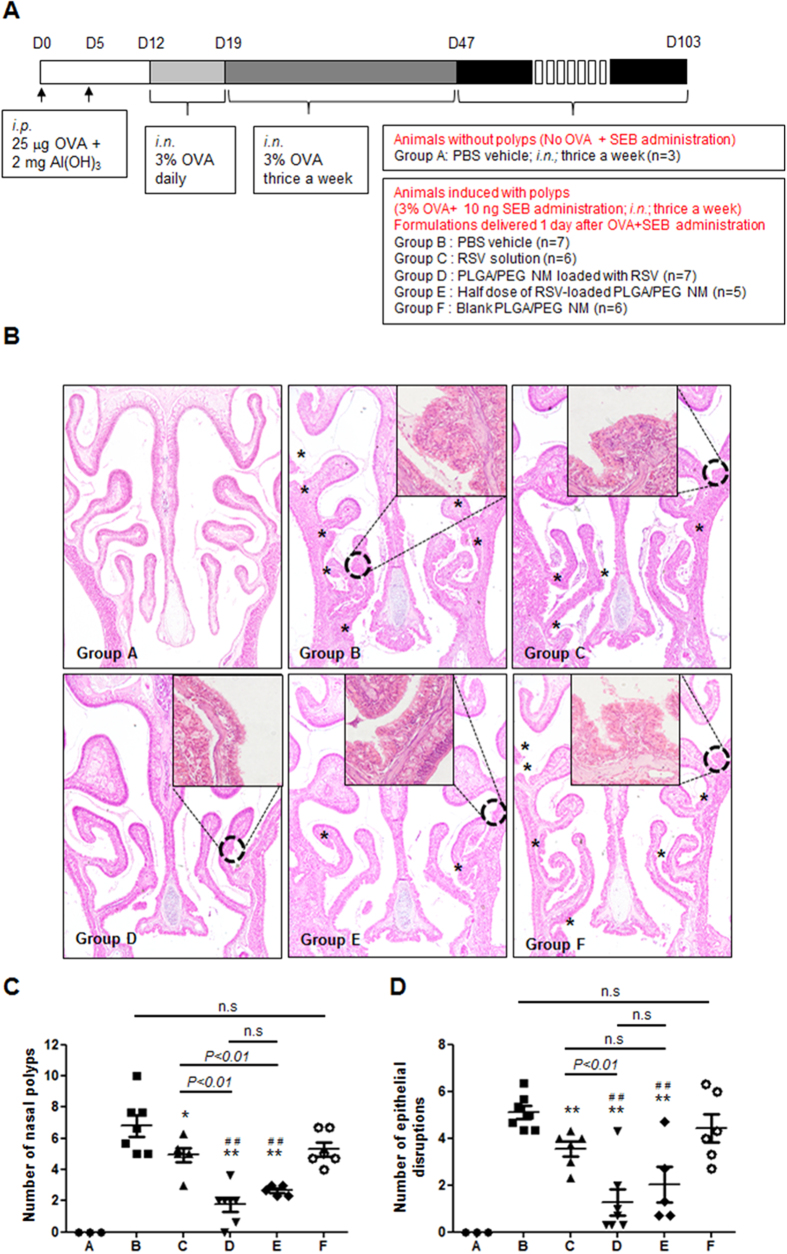
*In vivo* effects on nasal polyps and epithelial disruptions. (**A**) Schematic illustration of the procedure for generating the murine nasal polyp (NP) animal model. To induce nasal polyps, mice were immunized with an intraperitoneal (i.p.) injection of 25 μg ovalbumin (OVA) in 2 mg of aluminum hydroxide gel on days 0 and 5, followed by daily intranasal (i.n.) administrations of 40 μl of a 3% OVA solution in phosphate-buffered saline (PBS; pH 7.4) at days 12–19. Subsequently, 40 μl of a 3% OVA solution was delivered intransally 3 times per week until day 47. Finally, to maintain prolonged inflammation, 3% OVA solution (40 μl) and 10 ng of SEB (40 μl) were administered 3 times per week until the end point of the experiments. (**B**) Representative H&E-stained images of the posterior part of the nasal cavity. The inset, which is magnified from the dotted circle, shows a representative image of polyp formation. The asterisks indicate the locations of classical polypoid lesions. For each animal group, the numbers of (**C**) nasal polyps and (**D**) epithelial disruptions were counted and averaged in 3 distinct HPFs. Significant differences are denoted relative to Group B (PBS vehicle) (*P < 0.05, **P < 0.01) or to Group F (Blank PLGA/PEG NM) (^#^*P* < 0.05, ^##^*P* < 0.01).

Given the substantially improved intranasal retention and sustained drug release properties of PLGA/PEG NM, we sought to examine their *in vivo* efficacy as intranasal RSV delivery carriers. To do so, we applied the RSV-loaded PLGA/PEG NM to a previously established murine nasal polyp model (Fig. 3A)^5^,^6^,^10^,^25^. The polyp model was generated by chronic administration of ovalbumin (OVA) and *Staphylococcus aureus* enterotoxin B (SEB) (Groups B-F). Group A consisted of animals that did not receive this treatment, representing the polyp-free control group. After the OVA and SEB treatments, we administered the different RSV formulations to examine their effects on the polyps in the model animals.

We first examined the number of polyps in the sinonasal cavity. Group B was administered with the vehicle, i.e., PBS without RSV, and animals showed 5 or more nasal polyps in the sinonasal cavity. However, upon intranasal delivery of RSV solution (Group C), there was an apparent reduction in polyp number compared to that of Group B, in accordance with our previous results^6^. When the same amount of RSV was administered with PLGA/PEG NM (Group D), the number of polypoid lesions was further reduced compared to that of Group C (Fig. 3C). Notably, this reduction was also obvious with half the dose of RSV-loaded PLGA/PEG NM (also representing half the dose of RSV) (Group E). As expected, blank (no RSV) PLGA/PEG NM (Group F) showed almost no reduction in polypoid formation compared with Group B. We also counted the number of epithelial disruptions, as shown in Fig. 3D. The animals treated with the formulations containing RSV (Groups C, D and E) exhibited a decreased number of epithelial disruptions compared with Groups B and F. Again, the reduction was greatest with RSV-loaded PLGA/PEG NM (Groups D and E).

### *In Vivo* Evaluation of Inflammatory Surrogates

**Figure 4:**
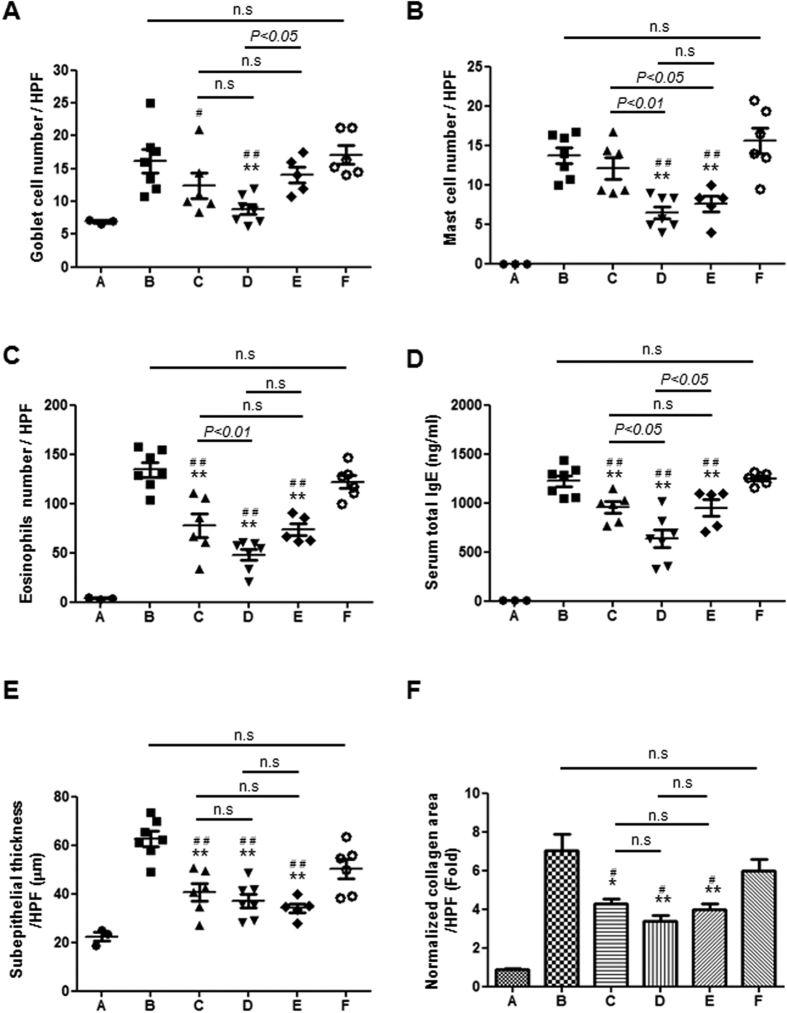
*In vivo* evaluation of inflammatory markers. For each animal group, the numbers of (**A**) goblet cells, (**B**) mast cells and (**C**) infiltrating eosinophils, (**D**) serum total IgE levels, (**E**) thickness of the subepithelial collagen layer and (**F**) areas of sub-epithelial collagen deposition were assessed and averaged from 3 distinct HPFs. Significant differences are denoted relative to Group B (PBS vehicle) (*P < 0.05, **P < 0.01) or to Group F (Blank PLGA/PEG NM) (^#^*P* < 0.05, ^##^*P* < 0.01).

Next, we examined the anti-inflammatory effect of RSV when delivered in the various formulations tested in this study. As shown in Fig. 4, in the absence of RSV (Groups B and F), prominent inflammation was observed in the nasal cavity and paranasal sinus. In contrast, the overall degree of inflammation was suppressed by administration of an RSV solution (Group C), while RSV-loaded PLGA/PEG NM (Groups D and E) showed an even greater effect. Again, even when of the dose of RSV delivered with PLGA/PEG NM was cut in half (Group E), its therapeutic effect on inflammation was comparable to that of the RSV solution (Group C), implying that the PLGA/PEG NM enhanced the therapeutic effect of sinonasally delivered RSV on inflammation. The numbers of both mast cells and goblet cells were prominently decreased in Group D (Fig. 4A and B). There was also a significant decrease in the number of mast cells, even with the half dose of RSV encapsulated with PLGA/PEG NM (Group E). As shown in Fig. 4C, we also assessed the number of eosinophils, which play a critical role in the pathogenesis of human and murine nasal polyps^26^. The number of eosinophils was markedly decreased in all of the RSV-treated groups (Groups C, D and E). Notably, the greatest reduction in the number of eosinophils was observed in Group D. While total serum IgE levels increased in response to the OVA and SEB administration (Fig. 4D), we observed that they decreased following RSV administration (Groups C, D and E). This effect was preserved with a half dose of RSV-loaded PLGA/PEG NM (Group E). As shown in Fig. 4E and F, treatment with OVA and SEB induced prominent collagen accumulation in Groups B and F. Meanwhile, Groups C, D and E, which were treated with RSV, exhibited reduced collagen accumulation, as reported in our previous study^27^. Importantly, this effect was still evident with a half dose of RSV when delivered with PLGA/PEG NM (Group E). Representative histological images are shown in Figure S5 of the Supplementary Information.

### *In Vivo* Efficacy on E-cadherin Restoration

**Figure 5:**
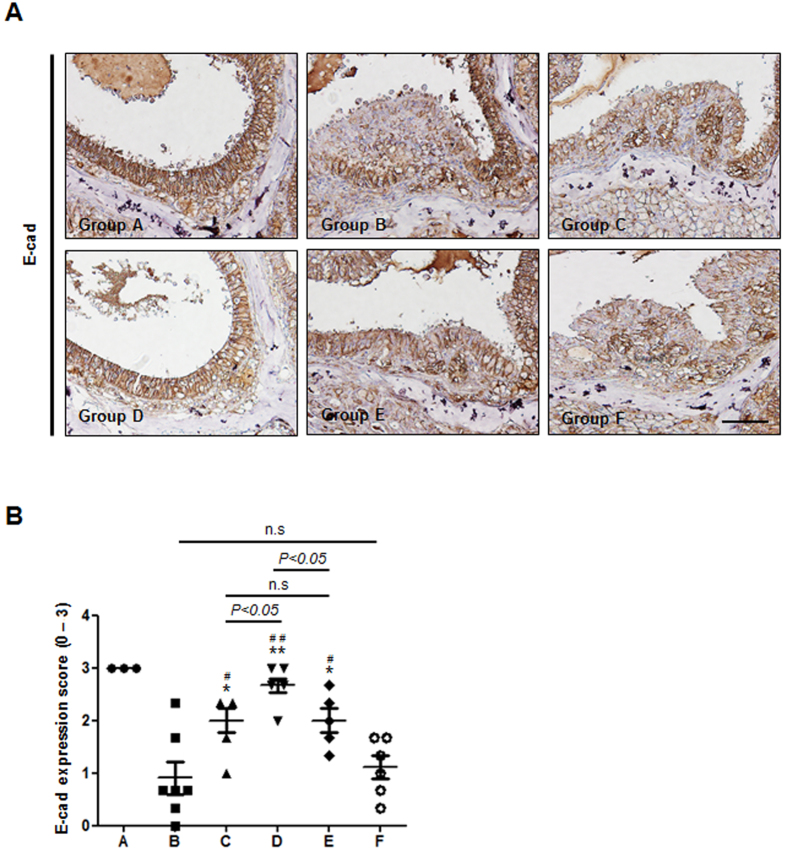
*In vivo* efficacy of attenuating HIF-1α activity. (**A**) Representative images of E-cadherin expression in the ethmoidal sinus mucosal epithelium. (**B**) Semiquantitative score plots of E-cadherin expression. Significant differences are denoted relative to Group B (PBS vehicle) (*P < 0.05, **P < 0.01) or to Group F (Blank PLGA/PEG NM) (^#^*P* < 0.05, ^##^*P* < 0.01).

RSV is known to activate SIRT1, which then attenuates HIF-1α activity in nasal epithelial cells through deacetylation^6^,^28^. In this work, we assessed E-cadherin expression as a representative marker of HIF-1α activity. As shown in Fig. 5A, E-cadherin loss in nasal epithelial cells was attributed to increased HIF-1α activity^5^. The nasal epithelium in Groups B and F showed markedly decreased E-cadherin expression compared to normal intact epithelium (Group A). However, upon administration of the RSV solution (Group C), E-cadherin expression remained high with respect to Groups B and F, indicating a marked reduction in polyp-formation capacity^6^. As anticipated, animals treated with RSV-loaded PLGA/PEG NM (Groups D and E) exhibited significantly greater E-cadherin expression. As in the other assays, a half dose of RSV-loaded PLGA/PEG NM still exhibited a notable result, blocking nasal epithelium remodeling as effectively as a full dose of the RSV solution. Even with continuous exposure to SEB and OVA, the RSV-loaded PLGA/PEG NM (Group D) could preserve the epithelium in nearly intact and healthy conditions resembling those in Group A (Fig. 5B).

### Biocompatibility Evaluation

**Figure 6:**
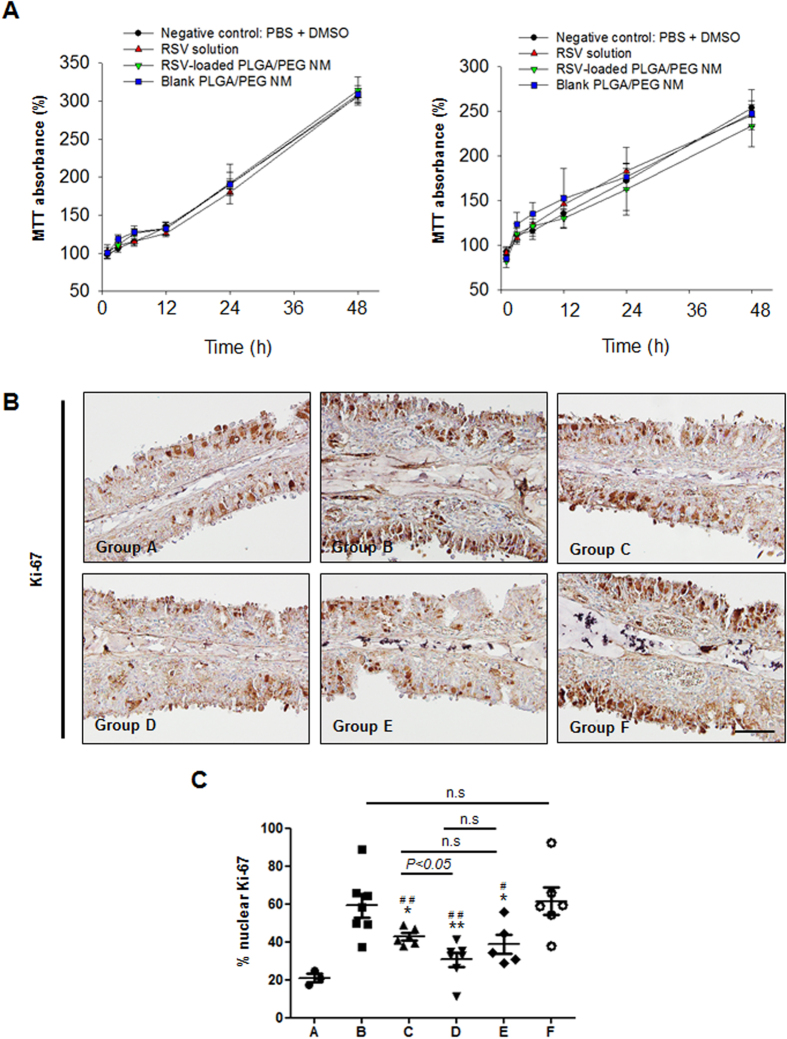
Evaluation of the biocompatibility of RSV-loaded PLGA/PEG NM. (**A**) The cytotoxicity of RSV, RSV-loaded PLGA/PEG NM and blank PLGA/PEG NM was evaluated with RPMI 2650 cells (nasal septum-derived squamous cell carcinoma) and with hNECs (human nasal epithelial cells). The relative cell viabilities at 48 h after treatment are expressed as percentages of the control (measured at 0 h). (**B**) Representative images of Ki-67 expression in the mucosal epithelium. (**C**) The numbers of Ki-67-positive nuclei within the nasal epithelium were counted in 3 different HPFs and averaged for each animal group. Significant differences are denoted relative to Group B (PBS vehicle) (*P < 0.05, **P < 0.01) or to Group F (Blank PLGA/PEG NM) (^#^*P* < 0.05, ^##^*P* < 0.01).

We first assessed the cytotoxicity of the formulations tested in this work using 2 different types of human nasal epithelial cells, i.e., nasal septum-derived carcinoma cells (RPMI 2650) and primary nasal epithelial cells (hNEC). As shown in Fig. 6A, the viability and proliferation of cells treated with RSV solution, RSV-loaded PLGA/PEG NM or blank PLGA/PEG NM were all similar to that of cells treated with a nontoxic PBS + DMSO solution^29^,^30^, suggesting that the tested formulations are not cytotoxic under the concentration employed in this work (Figure S6 in the Supplementary Information). For *in vivo* biocompatibility evaluation, we examined Ki-67, a marker for cell proliferation, in nasal mucosa samples obtained from the animals (Fig. 6B and C). Ki-67 is a nuclear protein that is ubiquitously expressed in the G1, S and G2 phases of the cell cycle but not in G0 phase^31^,^32^. Ki-67 expression was significantly increased relative to Group A in Groups B and F, which were not treated with RSV. However, with RSV administration (Groups B, C and D), Ki-67 expression was reduced to a level similar to that of Group A, suggesting that cell proliferation was properly maintained. This effect was most apparent with RSV-loaded PLGA/PEG NM (Group D), and the effect of a half dose of RSV-loaded PLGA/PEG NM was comparable to that of a full dose of RSV solution (Group C).

## Discussion

RSV, which is abundant in red wine, grapes and blueberries, is known as a SIRT1 activator, and it is renowned for its anti-inflammatory, anti-oxidant and anti-cancer effects^7^,^8^. Indeed, a large body of recent reports has verified the health-promoting bioactivity of RSV^33^,^34^,^35^,^36^,^37^,^38^. As one of its many effects, RSV has been reported to have a therapeutic effect on nasal polyps^6^,^39^. However, the administration of RSV is limited by its very low aqueous solubility^40^,^41^. Thus, when formulated in solution, the RSV concentration is so low that it is difficult for RSV to diffuse into the target tissue after administration. The RSV concentration can be increased by the use of an organic solvent as an additive in the formulation; however, this strategy may not be favorable for safety reasons^42^,^43^,^44^. Moreover, RSV in solution can be rapidly cleared from an administration site such as the sinonasal cavity, further hampering drug absorption and thereby resulting in very low drug bioavailability.

Therefore, most recent studies have counteracted the low aqueous solubility of RSV by formulating it in nanoparticles, thereby increasing the specific surface area contacting the surrounding medium^45^,^46^,^47^,^48^. Nanoparticles also have the advantage of enhanced penetration through such delivery barriers as skin^49^ or endothelial tissues^50^. However, the enhanced solubility of RSV may compensate for the relatively rapid release of RSV from the nanoparticles. In addition, previous approaches did not consider how well RSV was retained by carriers at the administration site, which is one of the important factors that determine local drug bioavailability.

The mucoadhesive nanostructured microparticles described in this study have the advantage of a long residence time at the administration site in the sinonasal cavity. Our findings showed that the presence of a mucoadhesive substance alone (as in PLGA/PEG MS) appeared to improve microparticle retention to some extent^51^,^52^; however, a marked improvement was observed when mucoadhesion was synergistically enhanced by the enlarged specific surface area of the nanostructures in the PLGA/PEG NM (Table 1 and Fig. 2). In addition to their improved adherence to the mucosal layer in the sinonasal space, the PLGA/PEG NM released RSV in a sustained manner (Fig. 1). After releasing 58.9% of the loaded RSV during the initial 6 h, RSV was slowly released at a rate of 1.14% per h throughout the half-life of PLGA/PEG NM (23.5 ± 4.7 h; Table 1).

Therefore, the PLGA/PEG NM reported here can better mitigate polyp formation in an *in vivo* mouse model of eosinophilic rhinosinusitis with nasal polyps. Our results showed a significant reduction in the number of nasal polyps with PLGA/PEG NM treatment compared with RSV solution treatment (Fig. 3). This therapeutic effect may be ascribed to the enhanced anti-inflammatory effect of RSV (Fig. 4) and to the more evident attenuation of HIF-1α expression due to higher SIRT1 activity (Fig. 5)^6^. Additionally, both the longer retention and sustained RSV-release properties of the PLGA/PEG NM in the sinonasal cavity likely contribute to the improved therapeutic effects of RSV. An improvement in local RSV bioavailability is further suggested by the persistence of the effect, even with delivery of a half dose of the RSV-loaded PLGA/PEG NM.

Another advantage of the PLGA/PEG NM reported here is their good biocompatibility. PLGA is known to be highly biocompatible; thus it has already been used in a wide range of biomedical applications, such as biodegradable sutures, skin substitutes, and bioabsorbable bone fixation devices^14^,^53^,^54^. For the same reason, PEG has also been employed as a constituent of biological formulations^55^ and has been widely researched as a bio-functional material^16^,^17^,^56^. In this work, we prepared PLGA/PEG NM by physically mixing the 2 biocompatible polymers. We attempted to enlarge the specific surface area of the PLGA/PEG NM to improve the mucoadhesive effect of the PEG additive. As the particles were of the order of microns in size, the PLGA/PEG NM would not be internalized by the cells, thereby minimizing the possibility of nanotoxicity^57^,^58^,^59^. Accordingly, the PLGA/PEG NM were not cytotoxic to nasal septa or epithelial cells in our tests (Fig. 6A). Cell proliferation in the nasal mucosa was properly maintained when it was treated with RSV-loaded PLGA/PEG NM (Fig. 6B).

## Methods

### Materials

Poly (lactic-co-glycolic acid) (PLGA; glycolic acid:lactic acid = 1:1, i. v. = 0.43 dl/g) and polyethylene glycol (PEG; MW = 6 kDa) were provided by Evonik Industries (Germany) and Acros Organics (NJ, USA), respectively. Resveratrol (RSV), polyvinyl alcohol (PVA; 87–89% hydrolyzed; MW = 31–50 kDa), mucin from porcine stomach (Type III), calcium chloride dehydrate, Tween 20, Tween 80 and dimethyl sulfoxide (DMSO) were obtained from Sigma (MO, USA), and diethylthiatricarbocyanine iodide (DTTCI) was purchased from Alfa Aesar (MA, USA). Dimethylformamide (DMF), dichloromethane (DCM), and tetrahydrofuran (THF) were obtained from Mallinckrodt (MO, USA), J. T. Baker (NJ, USA), and Daejung (Korea), respectively. Potassium phosphate monobasic (KH_2_PO_4_), sodium hydroxide pellets (NaOH), sodium chloride (NaCl) and potassium chloride (KCl) were purchased from Daejung (Korea), and acetonitrile (ACN) was obtained from J. T. Baker (NJ, USA). Phosphate buffered saline (pH 7.4) was obtained from the Seoul National University Biomedical Research Institute. Ovalbumin (OVA, grade V; Sigma, St. Louis, MO) were used to sensitize and challenge experimental mice. Staphylococcus aureus enterotoxin B (SEB, List Biological laboratories, INC, CA) were utilized to induce nasal polyp in sino-nasal cavity.

### Microparticle Preparation

Four distinct types of microparticles, namely PLGA MS, PLGA/PEG MS, PLGA NM and PLGA/PEG NM, were prepared according to a previously reported protocol^19^,^20^ with slight modifications. The microparticles were loaded with a drug, RSV, to examine their efficacy as sinonasal drug delivery carriers. Alternatively, a fluorescent dye, DTTCI, was loaded to evaluate the *in vivo* retention of microparticles in the sinonasal cavity. The details of the microparticle fabrication procedure are provided in the Supplementary Information.

### Microparticle Characterization

The microparticles were imaged under a scanning electron microscope (SEM; 7401F, JEOL, Japan). The size distribution of microparticles was assessed using a Coulter counter (Multisizer 4, Beckman Coulter, CA, USA), and at least 10,000 microparticles were counted for each sample type. The amount of PEG that was present in microparticles was determined using a proton ^1^H - nuclear magnetic resonance (^1^H NMR) spectrometer (Bruker spectra spin 500 MHz, Leipzig, Germany)^19^,^20^. The specific surface areas of the microparticles were measured using a surface area and porosity analyzer (TriStar II 3020, Micromeritics, GA, USA)^19^,^20^. Approximately 5–10 mg of microparticles was completely dissolved in DMF, and a spectrophotometer (UV-1800, Shimadzu, Japan) was used at 770 nm or 340 nm to measure the amounts of loaded DTTCI or RSV, respectively. The *in vitro* drug release experiments were performed with RSV-loaded microparticles in 1.5 ml of PBS (pH 7.4) containing 1% w/v Tween 20 at 37 °C. At scheduled times, the release medium was collected and assessed via high-performance liquid chromatography (HPLC; Agilent 1260 series, Agilent Technologies, CA, USA). The details of the HPLC measurement procedure are described in the Supplementary Information. An *in vitro* assay was performed to evaluate the mucoadhesive property of the PLGA/PEG NM, following the protocol established in the previous study^22^. For this, 8 mg of either PLGA NM or PLGA/PEG NM was suspended in 2 ml of 1 mg/ml mucin solution prepared in DI water (mucin from porcine stomach, Type III), which was then incubated at 37 °C for 30 min. After that, the suspension was centrifuged at 10,000× g for 10 min and the aliquot was measured spectrophotometrically, using the periodic acid/Schiff staining method^22^, to give the amount of non-adsorbed mucin. To calculate the amount of mucin adsorbed to the microparticles, the non-adsorbed mucin amount was subtracted from the mucin amount that was originally added in the solution. The experiments were performed in triplicate for statistics.

### Cytotoxicity Assay

The cytotoxicity of the RSV solution, the RSV-loaded PLGA/PEG NM and the blank PLGA/PEG NM was evaluated with RPMI 2650 (nasal septum-derived squamous cell carcinoma) and hNECs (human nasal epithelial cells) using a methyl thiazolyl tetrazolium (MTT) viability assay. The cells were seeded in 96-well culture plates at a density of 1 × 10^4^ cells per well in 200 μl of culture medium. The RPMI 2650 cells were cultured in RPMI 1640 medium supplemented with 10% FBS, 100 units/ml of penicillin and 100 μg/ml streptomycin. The hNECs were cultured in airway epithelial cell growth medium from PromoCell (Heidelberg, Germany). The cells were grown at 37 °C in a 5% CO_2_ humidified environment. After 12 h, the culture medium from each well was completely removed, and then 160 μl of fresh culture medium and 40 μl of the test medium were added. We employed 4 distinct testing media, namely RSV solution (0.88 μg RSV), RSV-loaded PLGA/PEG NM suspension (0.83 mg particles), blank PLGA/PEG NM suspension (0.83 mg particles) and a mixture of DMSO and PBS (1:450, v/v) (i.e., PBS + DMSO vehicle without RSV or PLGA/PEG NM). The RSV solution was prepared by dissolving 10 μg of RSV in a solution consisting of 1 μl of DMSO and 450 μl of PBS containing 0.02% v/v Tween 80. The microparticle suspensions were prepared in PBS containing 0.02% v/v Tween 80. After 12, 24, 36 and 48 h of incubation, the cells were washed twice and incubated in fresh culture medium mixed with MTT (1 mg/ml) for 3 h at 37 °C. Finally, the MTT solution was removed, and the cells were resuspended in 100 μl of 2-propanol to lyse the cells and completely dissolve the dye. The absorbance was measured at a wavelength of 570 nm using a Vmax microplate reader (Molecular Devices, USA). Triplicate experiments were conducted for each test medium and time. The cell viability was expressed as a percentage of the control (measured at 0 h).

### *In vivo* Sinonasal Retention of Microparticles

All procedures were performed under an animal protocol approved by the Institutional Animal Care and Use Committee of the Biomedical Research Institute at the Seoul National University Hospital (IACUC No. 13–0277-C3A2). This protocol complied with the NIH *Guide for* “*The Care and Use of Laboratory Animals*”. Male BALB/c nude mice (4 weeks old, Orient Bio, Korea) were used to assess the *in vivo* sinonasal retention of the microparticles. The mice were housed in standard cages in a well-controlled environment (temperature, 22 °C ± 2 °C; humidity, 50% ± 10%, ventilation, 12–18 times/h) with free access to food and water.

For this experiment, we used suspensions of DTTCI-loaded microparticles, which were sinonasally administered^60^. To prepare the suspensions, 1 mg of DTTCI-loaded microparticles were added to 40 μl of pH 7.4 PBS containing Tween 80 (0.02% v/v) as a dispersing agent, thus yielding a 25 mg/ml microparticle suspension. For each of the microparticle types, 20 μl of suspension was administered into each of the 2 nasal cavities in each mouse (i.e., a total of 40 μl for each mouse)^10^,^61^.

Immediately after administration, the mouse was placed under anesthesia using isoflurane and the whole head of each mouse was imaged using an IVIS (Caliper Life Sciences, USA) equipped with indocyanine green filters (excitation, 748–789 nm; emission, 814–851 nm) under the following conditions: exposure time, 1 s; binning factor, medium; f-stop, 4; and field of view, 10 × 10 cm. The intensity of the fluorescence signal from the nasal cavity at each time point was measured in the selected region of interest and was normalized to the maximum intensity (obtained at 2 h after administration). For each microparticle type, 3 animals were tested for statistical analysis.

### Animal Nasal Polyp Model

We prepared the animal nasal polyp model using 4-week old BALB/c mice (Central Laboratory Animal, Korea), as depicted in Fig. 3A, according to a slightly modified version of the protocol reported in our previous study^10^. The experimental protocol was approved by the IACUC of Seoul National University (IACUC No. SNU-150311-1) and complied with the NIH *Guide for “The Care and Use of Laboratory Animals*”. The animals were housed in specific pathogen-free (SPF) rooms with free access to food and water. In this work, we prepared a total of 34 animals and divided them into 6 distinct groups, A–F, according to the type of treatment. Group A consisted of animals without nasal polyps (i.e., no administration of OVA or SEB).

We prepared 5 different treatment formulations: PBS vehicle without RSV, a bolus RSV solution, and 3 distinct suspensions of PLGA/PEG NM in PBS. The formulations were intranasally administered 1 day after the OVA and SEB administration, which were scheduled 3 times per week. At each administration, 20 μl of the same formulation was applied to both nostrils; hence, a total of 40 μl was administered to each mouse. The 6 animal groups and treatment formulations were as follows:


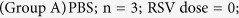



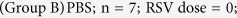



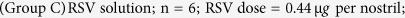



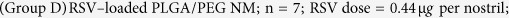



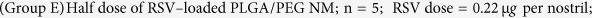



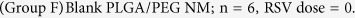


To prepare the RSV solution for Group C, 10 μg of RSV was dissolved in 1 μl of DMSO and was then added to 450 μl of PBS (pH 7.4). To prepare the microparticle suspensions for Groups D and E, 0.83 mg of PLGA/PEG NM with and without RSV were suspended in 40 μl of PBS at pH 7.4 and containing 0.02% w/v Tween 80 as a dispersing agent. For Group E, a half portion (0.415 mg) of PLGA/PEG NM loaded with RSV was suspended in 40 μl of PBS.

### Histological Analysis

Mice were euthanized and decapitated. Next, the skin on the head was stripped and the mandibles were excised. The tissue specimens were fixed in 4% paraformaldehyde and decalcified in 5% nitric acid for 4–5 days at 4 °C. The tissue was dehydrated and processed according to standard paraffin-embedding procedures. The tissues were stained with hematoxylin and eosin (H&E), Sirius red, Giemsa, Masson’s trichrome and PAS to assess overall inflammation, eosinophils, mast cells, collagen deposition and goblet cell hyperplasia, respectively. An atlas of normal murine sinonasal anatomy was used to standardize the anatomic locations being examined. Three coronal sections that were similar to the sinus cavity were chosen for evaluation. The sections were examined under a light microscope (x400, HPF; BX-51; Olympus, Japan) equipped with a camera (DP70, Olympus, Japan) by 2 independent examiners who were blinded to the experimental groups; the examiners determined the numbers of inflammatory cells and secretory cells and evaluated subepithelial collagen thickness and mucosal lesions, including polyps and epithelial disruptions. We defined a polypoid lesion as a distinct mucosal elevation with eosinophilic infiltration and microcavities. Epithelial disruptions were only counted if the pieces of epithelium were more than 50 μm in length and were stripped off of the epithelial regions or split from adjacent epithelial cells. Three consecutive slides were examined to minimize processing errors and to confirm the presence of mucosal lesions. Inflammatory cells, secretory cells, and subepithelial collagen thickness were counted or measured in mucosal samples in 10 high-power fields (HPFs). The numbers of inflammatory and secretory cells were expressed as cells per HPF. Subepithelial collagen thickness was quantified using ImageJ software (National Institutes of Health, Bethesda, Maryland, USA).

### Immunohistochemistry for E-cadherin Expression

Paraffin sections of the posterior mouse nose were rehydrated and microwave-treated in citrate buffer (pH 6.0) for antigen retrieval. The sections were treated with 3% hydrogen peroxide for 10 min and then incubated with rabbit anti-mouse E-cadherin (1:200; BD, NJ, USA) or goat anti-mouse Ki-67 antibodies (1:500; Santa Cruz Biotechnology, TX, USA) for 1 h at room temperature. The immuno-complexes were amplified using an HRP-conjugated secondary antibody and then visualized with a diaminobenzidine (DAB) detection system (Dako, California, USA). Finally, the slide was counterstained with Mayer’s hematoxylin. To semiquantitatively analyze E-cadherin expression, 3 different spots in each HPF were randomly selected and then assessed by 2 independent examiners who were blinded to the experimental group. When more than 30 continuous E-cadherin-positive epithelial cells were present, the spot was counted; thus, each HPF was scored from 0–3. For each animal group, the average score was obtained from 3 different HPFs (x400). Additional details about the methods are described in our previous report^6^.

### ELISA for Mouse Serum Total IgE Levels

Blood was collected via cardiac puncture and was then centrifuged at 1,300 rcf for 20 min. The obtained sera were stored at −70 °C until measurement. The total IgE levels in mouse serum were quantified using the LEGEND MAX ELISA kit from Biolegend (USA). A TMB (3,3′,5,5′-tetramethyl benzidine) substrate reagent was used to detect the antibody (San Diego, CA). Each assay was performed in duplicate and was measured spectrophotometrically at a wavelength of 450 nm using a Vmax microplate reader (Molecular Devices, USA).

### Statistical Analysis

The mean fraction of microparticles remaining in the sinonasal cavity was analyzed using analysis of variance (ANOVA) with α = 0.05, where pairwise comparisons were made using Tukey’s post hoc test. For the other statistical analyses, 2-tailed Mann-Whitney U tests were performed with IBM SPSS 21 (SPSS, USA) for unpaired comparisons. Figures were generated with GraphPad Prism software 6.0 (GraphPad Software, USA). Kruskal-Wallis tests were used for comparisons between pairs of groups to establish significant intergroup variability, and then 2-tailed Mann-Whitney U tests were performed for between-group comparisons. *P* < 0.05 was considered a significant difference.

## Additional Information

**How to cite this article**: Lee, M. *et al*. Sinonasal Delivery of Resveratrol via Mucoadhesive Nanostructured Microparticles in a Nasal Polyp Mouse Model. *Sci. Rep.* **7**, 40249; doi: 10.1038/srep40249 (2017).

**Publisher's note:** Springer Nature remains neutral with regard to jurisdictional claims in published maps and institutional affiliations.

## Supplementary Material

Supplementary Information

## Figures and Tables

**Table 1:** Half-lives of microparticles administered to the sinonasal cavity.

Microparticle type	Half-life (h)†
PLGA MS	10.5 ± 2.8
PLGA/PEG MS	9.1 ± 0.3
PLGA NM	21.5 ± 2.5
PLGA/PEG NM	23.5 ± 4.7

^†^Half-life was measured beginning 2 h after microparticle administration.
