# Supplementary material for: Sinonasal Delivery of Resveratrol via Mucoadhesive Nanostructured Microparticles in a Nasal Polyp Mouse Model
Source: Sci Rep. 2017 Jan 10;7:40249. doi: 10.1038/srep40249 (PMC5223156; doi:10.1038/srep40249)
Supplement: Supplementary Information [file srep40249-s1.pdf]

Supplementary Information

Sinonasal Delivery of Resveratrol via Mucoadhesive Nanostructured Microparticles in a Nasal Polyp Mouse Model

Mingyu Lee[1, 2, †], Chun Gwon Park[3, †], Beom Kang Huh[4], Se-Na Kim[4], Seung Ho Lee[3], Roza Khalmuratova[1], Jong-Wan Park[1, 2, 5, 6], Hyun-Woo Shin[1, 2, 5, 6, 7, *], and Young Bin Choy[3, 4, 8, *]

[1]Obstructive Upper airway Research (OUaR) Laboratory, Department of Pharmacology, Seoul National University College of Medicine, Seoul, 03080, Republic of Korea

[2]Department of Biomedical Science, Seoul National University Graduate School, Seoul, 03080, Republic of Korea

[3]Institute of Medical & Biological Engineering, Medical Research Center, Seoul National University, Seoul, 03080, Republic of Korea.

[4]Interdisciplinary Program in Bioengineering, College of Engineering, Seoul National University, Seoul, 08826, Republic of Korea.

[5]Ischemic/Hypoxic Disease Institute, [6]Cancer Research Institute, Seoul National University College of Medicine, Seoul, 03080, Republic of Korea

[7]Department of Otorhinolaryngology-Head and Neck Surgery, Seoul National University Hospital, Seoul, 03080, Republic of Korea

[8]Department of Biomedical Engineering, Seoul National University College of Medicine, Seoul, 03080, Republic of Korea.

[†]These co-first authors contributed equally to this work .

[*]To whom all correspondence should be addressed:

Corresponding author (Prof. Young Bin Choy)

E-mail: ybchoy@snu.ac.kr

Tel: + 82-2-740-8592; Fax: +82-2-741-6303

Corresponding author (Prof. Hyun-Woo Shin)

E-mail: charlie@snu.ac.kr

Tel: + 82-2-740-8285; Fax: +82-2-745-7996

Preparation of Microparticles. To prepare the spherical microparticles, i.e., PLGA MS or PLGA/PEG MS, 1,000 mg of PLGA or a blend of 1,000 mg of PLGA and 250 mg of PEG was dissolved in 10 ml of dichloromethane (DCM), respectively, into which 3 mg of resveratrol (RSV) or 10 mg of diethylthiatricarbocyanine iodide (DTTCI) was dissolved. The prepared polymer solution was then added to 50 ml of a polyvinyl alcohol (PVA) solution (1% w/v) and agitated vigorously at 1,000 rpm for 2 min for emulsification. The resulting emulsion was added to 50 ml of a PVA solution (1% w/v), which was then stirred at 200 rpm for 30 min under vacuum (-12.5 psi) for solvent evaporation. A 100 μm-pore metallic mesh (C8.20047, Chung Gye, Korea) was used to filter the resulting microparticles, which were then thoroughly washed with deionized (DI) water and freeze-dried.

To prepare nanostructured microparticles, i.e., PLGA NM or PLGA/PEG NM, 1,000 mg of PLGA or a mixture of 1,000 mg of PLGA and 100 mg of PEG, respectively, were dissolved in 3.35 ml of a solvent mixture of DCM, tetrahydrofuran (THF) and dimethylformamide (DMF) (3:1:1, v/v/v) in which either 1 mg of RSV or 8.5 mg of DTTCI had been dissolved. The solution was then electrospun for 1 h under the following conditions (Nano NC, Korea): applied voltage, 20 kV; tip-to-collector distance, 10 cm; collector rotation speed, 100 rpm; needle gauge, 26 G; and flow rate, 2.0 $ml \cdot h^{-1}$. The resulting nanofibrous sheets were then freeze-milled at -196°C for 30 min (6770 Freezer Mill, Spex, Metuchen, NJ, USA) and then filtered through a 100 μm-pore metallic mesh (C8.20047, Chung Gye, Korea).

HPLC measurement. The RSV concentration was measured with high-performance liquid chromatography (HPLC; Agilent 1260 series, Agilent Technologies, CA, USA) using a Diamonsil

C18 column (150 × 4.6 mm, 5 μm pore; Dikma Technologies, CA, USA). A mobile phase was prepared by mixing 20 mM phosphate-buffered saline (pH 2.5) and acetonitrile (60:40, v/v). The flow rate and injection volume were 1 ml/min and 30 μl, respectively. The column temperature was maintained at 37°C, and UV absorbance was measured at 333 nm.

Table S1. Properties of the DTTCI-loaded microparticles used in this work.

Microparticle type	Mean size (μm)	PEG content (wt %)	Specific surface area (m^2/g)	DTTCI content (μg/mg)
PLGA MS	7.46 ± 1.32	0	N/D	8.87 ± 0.03
PLGA/PEG MS	7.64 ± 1.22	9.95	28.88	8.27 ± 0.11
PLGA NM	7.44 ± 0.98	0	N/D	8.16 ± 0.08
PLGA/PEG NM	6.72 ± 1.12	10.17	291.23	7.93 ± 0.05

N/D, not determined

Table S2. Properties of the RSV-loaded microparticles used in this work.

Microparticle Type	RSV Content (μg/mg)
PLGA MS	1.78 ± 0.10
PLGA/PEG MS	1.10 ± 0.03
PLGA NM	1.62 ± 0.03
PLGA/PEG NM	1.06 ± 0.02

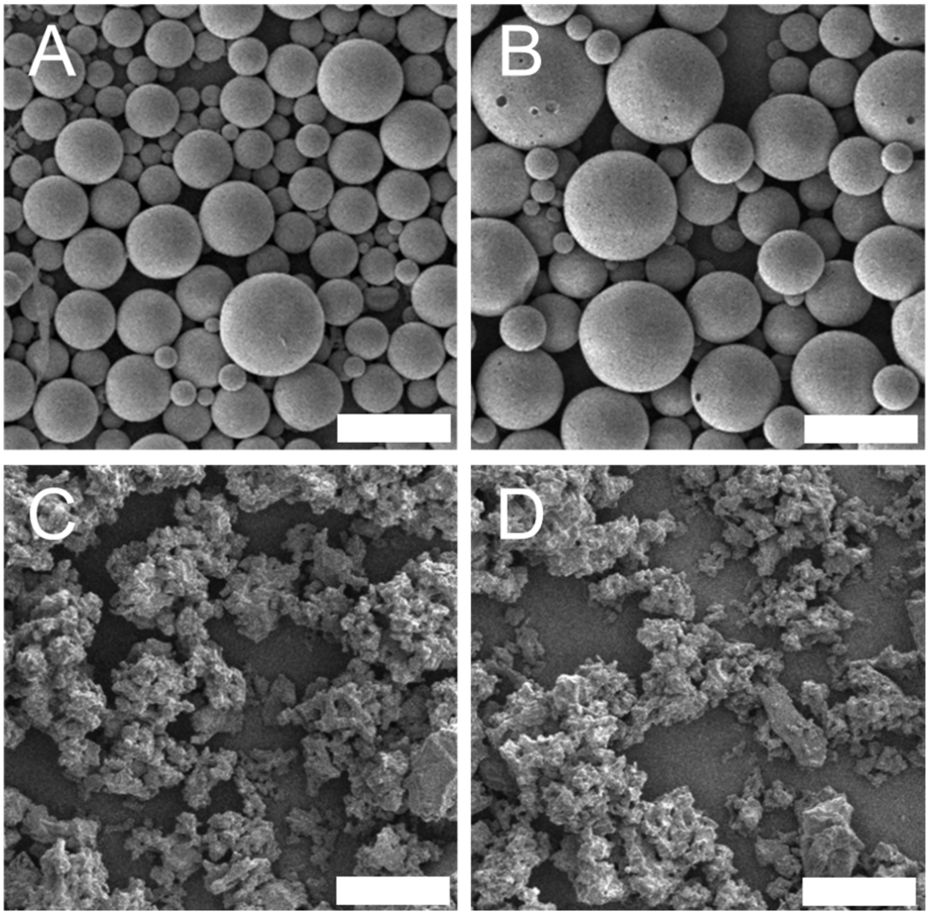

Figure S1. Representative scanning electron micrographs of (A) PLGA MS, (B) PLGA/PEG MS, (C) PLGA NM and (D) PLGA/PEG NM. The scale bars represent 50 µm.

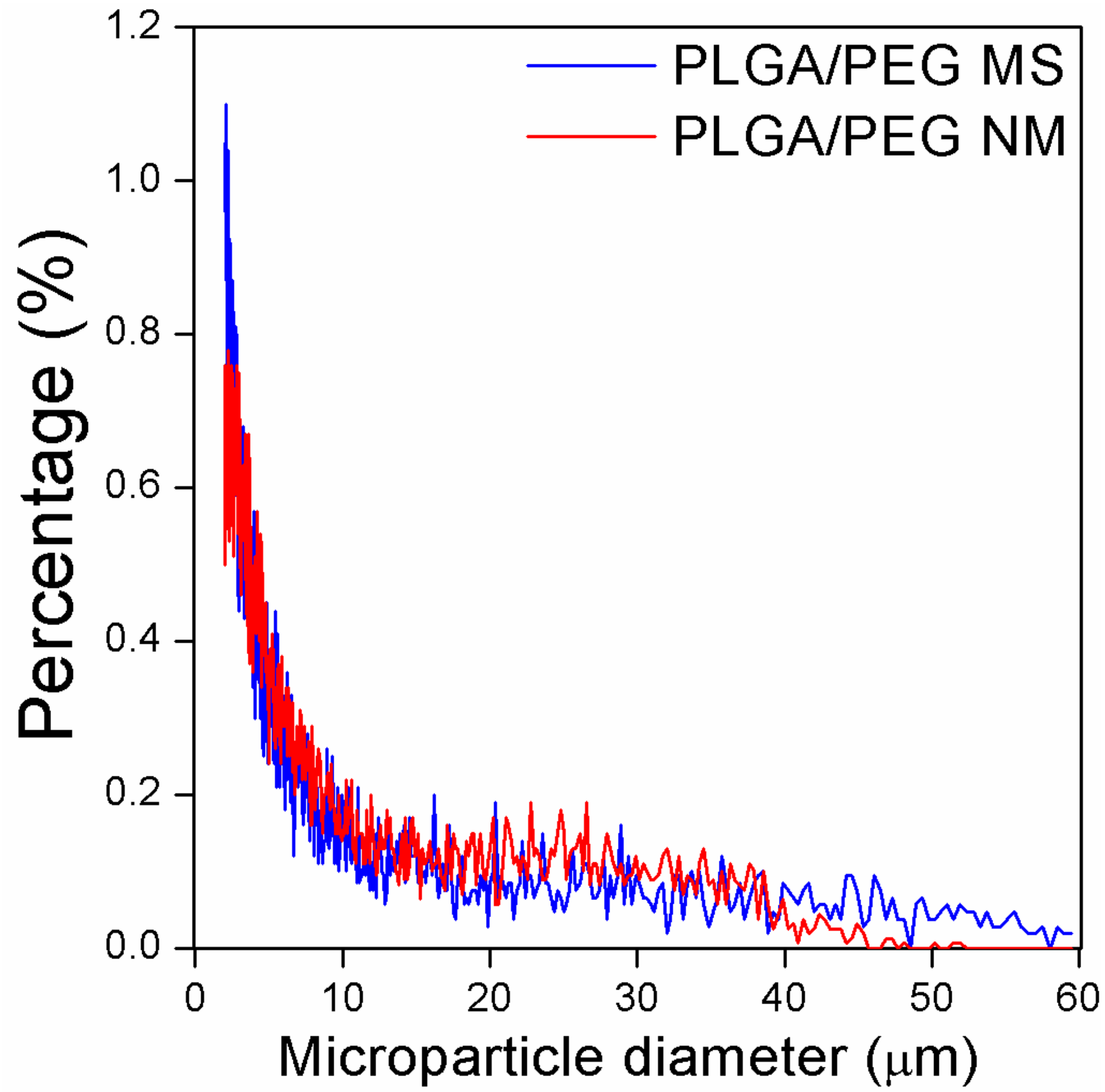

Figure S2. Size distribution profiles of PLGA/PEG MS (blue) and PLGA/PEG NM (red) as measured with a Coulter counter.

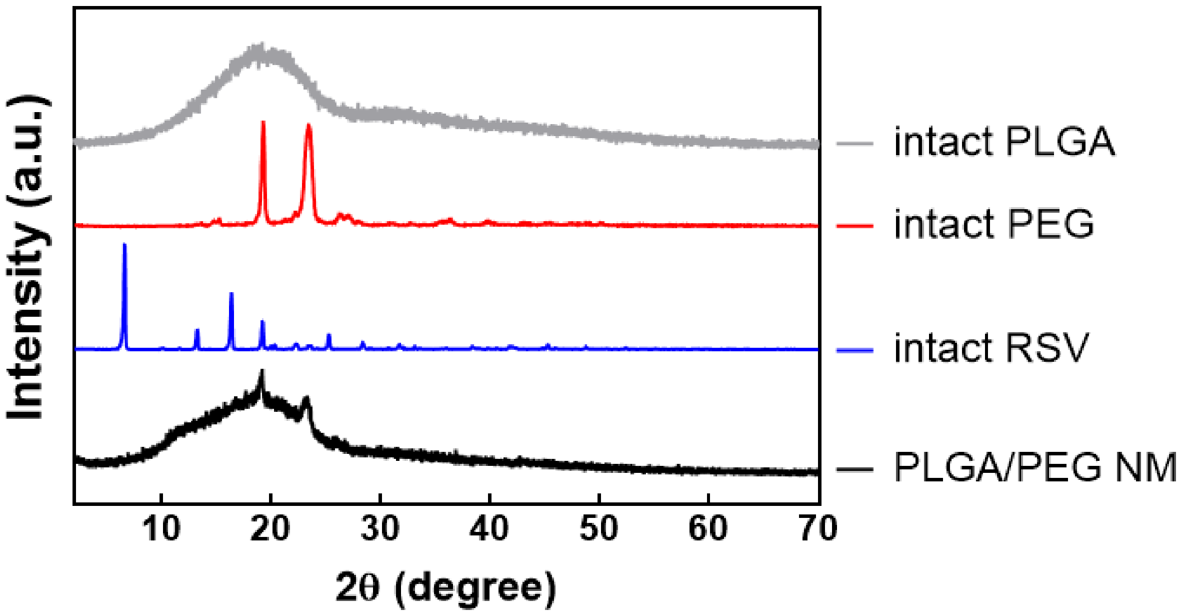

Figure S3. Powder X-ray diffraction (PXRD) patterns of intact PLGA, intact PEG, intact RSV and RSV loaded PLGA/PEG NM. The measurement was performed with an X-ray diffractometer (D/MAX RINT 2200-Ultima, Rigaku, Japan) that is equipped with Ni-filtered CuK_{α} radiation (λ = 1.5418 Å). Each sample was deposited on a glass substrate and scanned continuously at a tube voltage and current of 40 kV and 30 mA, respectively. For intact PEG and RSV, distinct crystalline peaks were observed[1,2] while intact PLGA did not exhibit any apparent peaks due to its amorphous structure.[3] For the RSV loaded PLGA/PEG NM, the characteristic peaks originated from RSV were not observed but the distinct peaks originated from PEG were seen. This result suggested that unlike PEG, RSV was distributed in the PLGA/PEG NM without forming a crystalline structure.

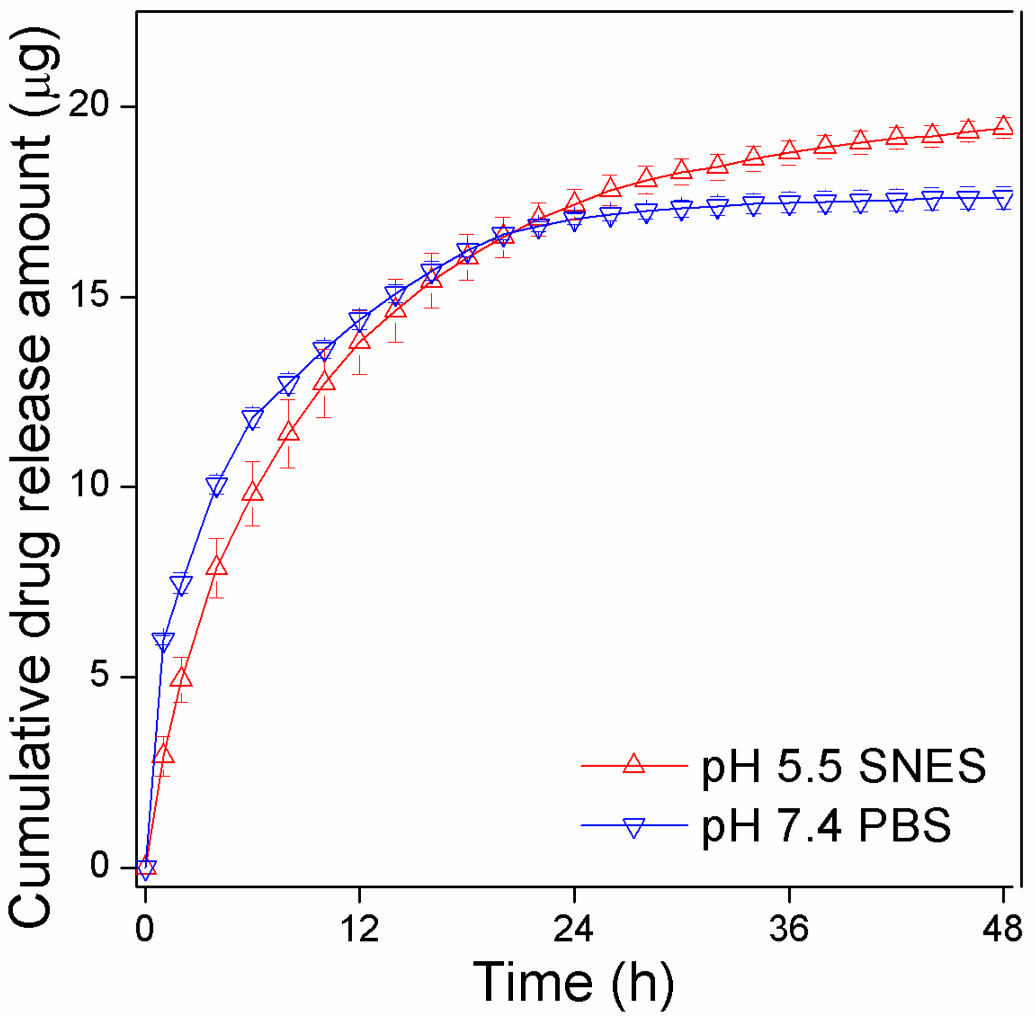

Figure S4. *In vitro* release profiles of RSV from the PLGA/PEG NM obtained with pH 5.5 simulated nasal electrolyte solution containing 1% w/v Tween 20. At defined time points, the release medium was collected and measured by HPLC, as depicted in the Methods section. The release profile obtained in pH 7.4 PBS (Fig. 1) was replotted in the same graph for comparison. An overall release profile of RSV was not significantly different but a slightly higher release was observed at pH 5.5 compared with at pH 7.4. At pH 5.5, RSV is known to be more stable[4] and thus, more effective RSV appeared to be available in the release medium.

A

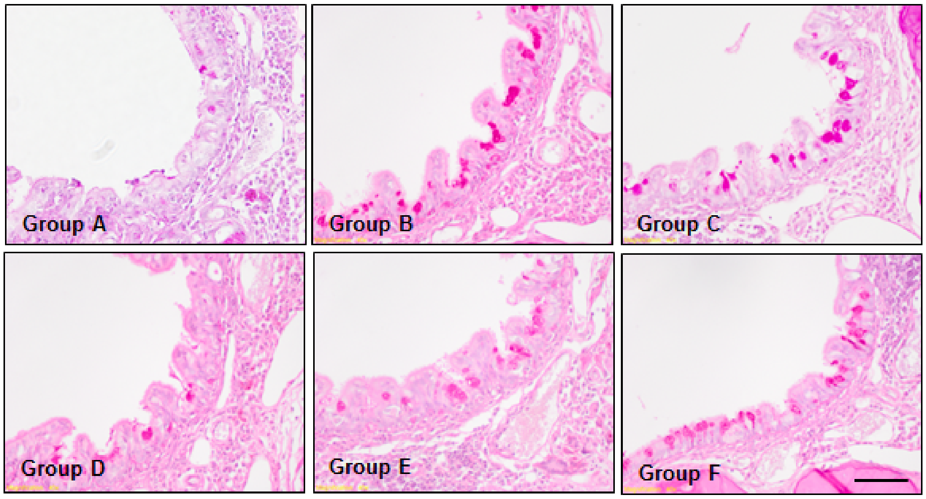
Group A
Group B
Group C
Group D
Group E
Group F

B

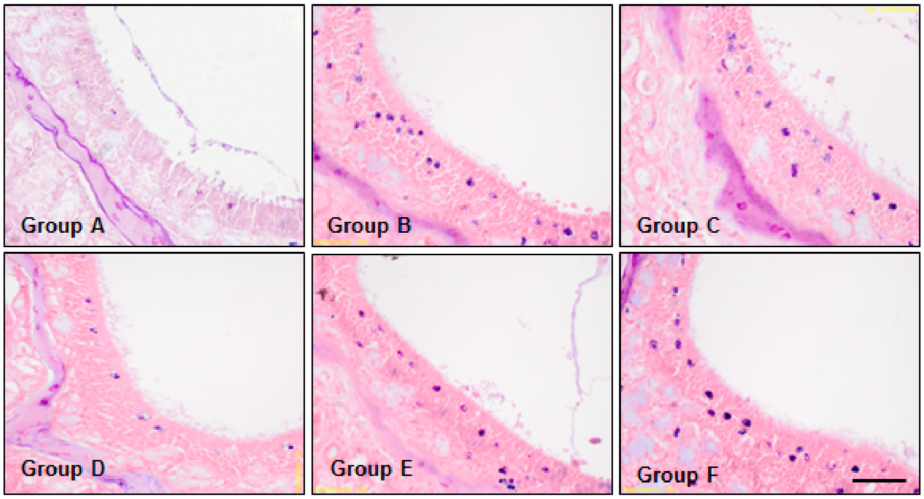
Group A
Group B
Group C
Group D
Group E
Group F

C

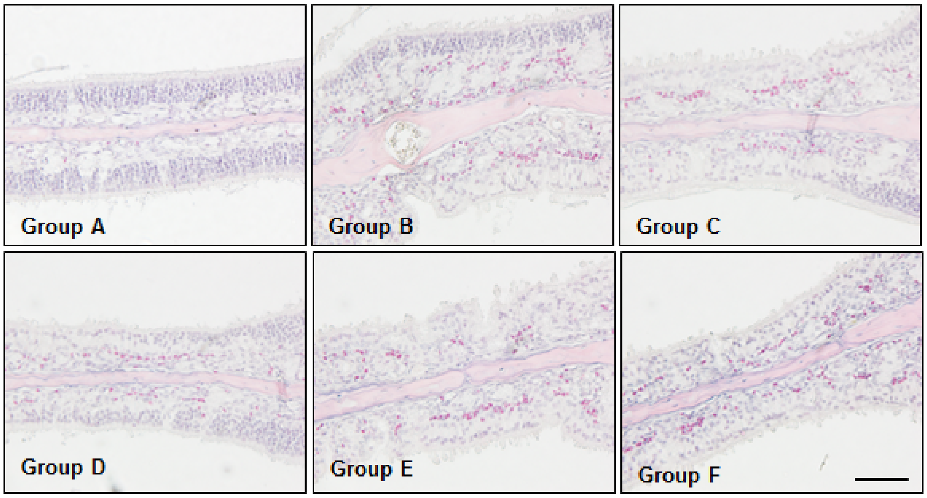

D

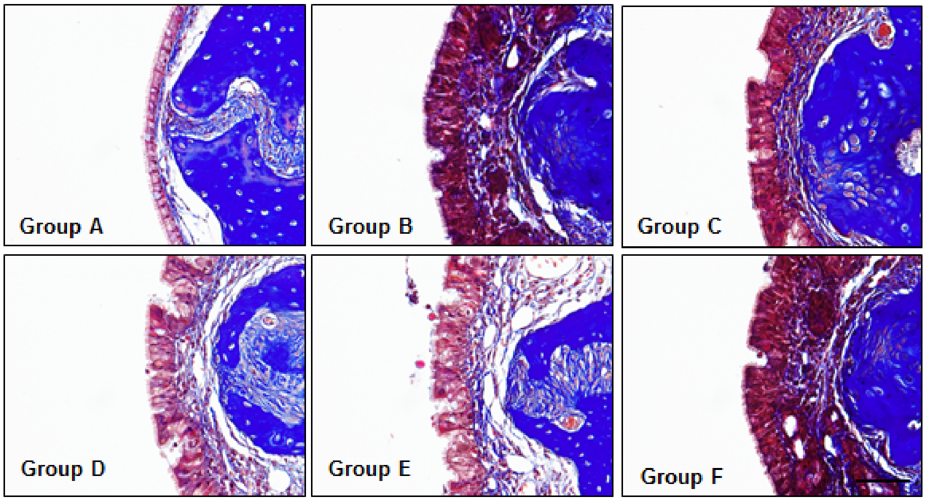

Figure S5. Representative stained sinonasal cavity specimens that were used to evaluate (A) goblet cells, (B) mast cells, (C) eosinophils, and (D) collagen deposition.

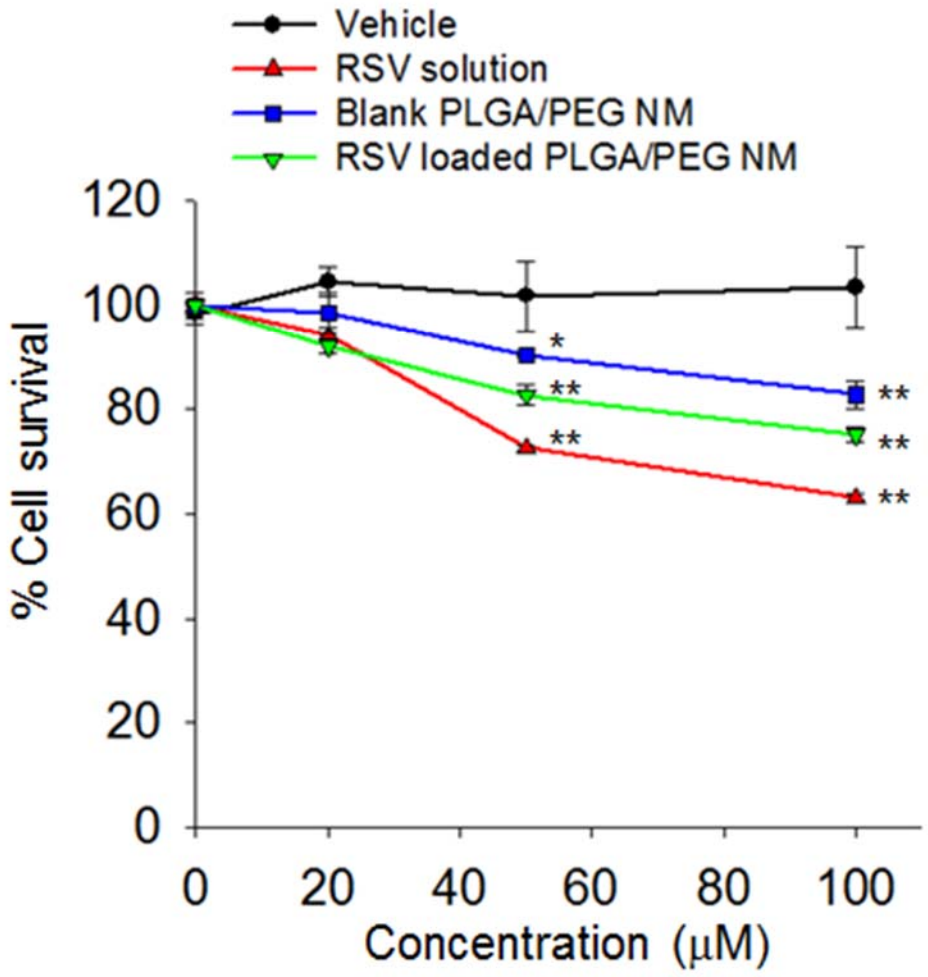

Figure S6. Evaluation of the biocompatibility of RSV-loaded PLGA/PEG NM at varied concentrations. The cytotoxicity was evaluated with RPMI 2650 cells (nasal septum-derived squamous cell carcinoma). For this, we prepared the formulations at the concentrations equivalent to 20, 50 and 100 μM RSV. Thus, to prepare the RSV solution, 0.88, 2.2 and 4.4 μg RSV was dissolved in 40 μl of a mixture of DMSO and PBS (1:450, v/v), respectively. To prepare the microparticle suspension, 0.83, 2.08 and 4.15 mg blank or RSV loaded PLGA/PEG NM was suspended in 40 μl PBS containing 0.02% v/v Tween 80, respectively. After treatment, cells were incubated for 48 h and treated with the MTT solution, as described in Methods. Triplicate experiments were conducted for each test medium. The cell viability was expressed as a percentage of the control (measured at 0 h).Significant differences are denoted relative to vehicle (PBS+DMSO vehicle) (*$P < 0.05$, **$P < 0.01$). The viability of cells decreased as the equivalent concentration of RSV increased[5-7], which was also observed with the blank PLGA/PEG NM. As compared with the RSV solution, the decrease in cell viability was less apparent with RSV loaded PLGA/PEG NM possibly due to slow release of RSV from the microparticles. Although a high concentration from 50 μM appeared to affect the cell viability, the equivalent RSV concentration at 20 μM exhibited that RSV loaded PLGA/PEG NM were not cytotoxic and under this condition, we performed all experiments depicted in this work.

Reference

1 Bolourchian, N., Mahboobian, M. M. & Dadashzadeh, S. The effect of PEG molecular weights on dissolution behavior of simvastatin in solid dispersions. *Iran J Pharm Res* **12**, 11-20 (2013).

2 Lee, C.-W. *et al.* Resveratrol nanoparticle system improves dissolution properties and enhances the hepatoprotective effect of resveratrol through antioxidant and anti-inflammatory pathways. *J. Agric. Food Chem.* **60**, 4662-4671 (2012).

3 Lee, J. E. *et al.* Surgical suture assembled with polymeric drug-delivery sheet for sustained, local pain relief. *Acta Biomater* **9**, 8318-8327 (2013).

4 Robinson, K., Mock, C. & Liang, D. Pre-formulation studies of resveratrol. *Drug Dev Ind Pharm* **41**, 1464-1469 (2015).

5 Bernhard, D. *et al.* Enhanced MTT-reducing activity under growth inhibition by resveratrol in CEM-C7H2 lymphocytic leukemia cells. *Cancer letters* **195**, 193-199 (2003).

6 Kotha, A. *et al.* Resveratrol inhibits Src and Stat3 signaling and induces the apoptosis of malignant cells containing activated Stat3 protein. *Mol. Cancer Ther.* **5**, 621-629 (2006).

7 Kang, J. H., Park, Y. H., Choi, S. W., Yang, E. K. & Lee, W. J. Resveratrol derivatives potently induce apoptosis in human promyelocytic leukemia cells. *Exp. Mol. Med.* **35**, 467-474 (2003).
